# Dietary effect of soybean lecithin on the growth performance, digestive enzyme activity, blood biomarkers, and antioxidative status of striped catfish, *Pangasianodon hypophthalmus*

**DOI:** 10.1371/journal.pone.0291954

**Published:** 2023-10-05

**Authors:** Abdel-Rahman Amer, Nabil M. Eweedah, Asem A. Amer, Mahmoud S. Gewaily, Nehal A. Younis, Hamada A. Ahmed, Mahmoud A. O. Dawood

**Affiliations:** 1 Faculty of Agriculture, Department of Animal Production, Kafrelsheikh University, Kafrelsheikh, Egypt; 2 Agriculture Research Center, Central Laboratory for Aquaculture Research, Abbassa, Sharkia, Sakha Aquaculture Research Unit, Kafrelsheikh, Egypt; 3 Faculty of Veterinary Medicine, Department of Anatomy and Embryology, Kafrelsheikh University, Kafrelsheikh, Egypt; 4 Faculty of Veterinary Medicine, Aquatic Animal Medicine and Management, Cairo University, Giza, Egypt; 5 Faculty of Veterinary Medicine, Department of Nutrition and Veterinary Clinical Nutrition, Damanhour University, Damanhour, Egypt; 6 The Center for Applied Research on the Environment and Sustainability, The American University in Cairo, Cairo, Egypt; Benha University, EGYPT

## Abstract

Soybean lecithin (SBL) is usually added to aquafeed as a lipid source because aquatic animals cannot synthesize phospholipids. Hence, this study aimed to investigate the role of SBL on the growth, nutrient consumption, digestive enzyme activity, blood parameters, and antioxidant capability of striped catfish. The fish were fed on five experimental diets with five grading levels of SBL (0, 2, 4, 6, and 8%) for 60 days. The final weight, weight gain, specific growth rate, feed intake, and protein efficiency ratio were markedly higher in striped catfish treated with 2–4% SBL than the control level (0% SBL). However, the lowest feed conversion ratio was in the fish-fed groups of 4–6% SBL. The carcass lipid content was significantly higher in fish fed 2–4% SBL compared to the control level (0% SBL). The lipase, amylase, and protease activities were significantly increased in the fish fed 2–6% SBL compared to 0% SBL-fed group. The gradually increased levels of SBL improved the structural appearance and increased the intestinal villi length and branching appearance. The triglycerides and total cholesterol were increased in the fish fed with 4, 6, and 8% compared to the control level, with the highest being in the fish fed with 8%. The lysozyme activity was higher in the fish fed with 2, 4, and 6% of SBL compared to the control level, with higher activity in the fish fed with 2 and 4% than 6%. Superoxide dismutase, glutathione peroxidase, and catalase activities were increased in the fish fed with 2, 4, and 6% SBL. The malondialdehyde level was lower in the fish fed with 4–6% SBL compared to the control level. The regression analysis revealed that the optimum dose of SBL is required at 3.65–4.42% for better productivity and health performances in striped catfish.

## 1. Introduction

Striped catfish (*Pangasianodon hypophthalmus*) have been introduced to the aquaculture industry in several countries due to their high productivity and suitability for consumers [1]. Striped catfish represent 4.3% of total finfish production globally [2]. Striped catfish is initially grown in the Mekong Delta of Vietnam and then spread to several Asian countries, making it more tolerant of stressful farming conditions [3]. Further, striped catfish can grow under intensive farming conditions without interrupting their growth performance and health status [4]. In order to have feasible productivity and profit, their nutritional needs must be met [5]. Thus, more efforts are still recommended to ensure the nutritional requirements of striped catfish [6]. Under the current circumstances of the high cost of ingredients which limit the profitability of finfish farming, it becomes necessary to formulate high-quality feeds with reasonable prices without affecting the nutritional value of fish feeds [7–9].

Soybean lecithin (SBL) is a natural feed ingredient known for its high content of phospholipids [10]. SBL is usually added to aquafeed as a lipid source because aquatic animals cannot synthesize phospholipids [11]. Thus, the exogenous source of phospholipids is recommended for inclusion in finfish feed to ensure the crucial needs for physiological and metabolic function [12,13]. Lecithin contains fatty acids, phosphate, and choline, which facilitate the metabolism of carbohydrates and lipids [14]. Phospholipids are involved in the structure of cell membranes and lipoproteins, which are needed for embryonic and larval growth and the luminal emulsification of lipids components [15,16]. Accordingly, lecithin can facilitate the absorption of lipids and digested nutrients through the intestines of fish [17,18]. The efficacy of SBL has been evaluated in several fish species. Including SBL led to improved physiological responses and pre-spawning capacity of Caspian brown trout (*Salmo trutta caspius*) [19]. Saleh et al. [20] stated that SBL enhanced the growth rate, regulated the fatty acids profile, and activated the antioxidant capacity of gilthead seabream (*Sparus aurata*). In Nile tilapia (*Oreochromis niloticus*), dietary SBL enhanced the growth performance, intestinal absorption capacity, and immune response [21] and successfully replaced fish meal and fish oil [22]. Interestingly, dietary SBL enhanced the growth performance and digestibility of grouper (*Epinephelus lanceolatus*) fed soybean-based diets [23]. Further, dietary phospholipids enhanced growth performance, regulated blood biomarkers, and promoted immunity and antioxidative responses in rainbow trout (*Oncorhynchus mykiss*) [18].

As indicated above, numerous beneficial roles were shown due to the inclusion of SBL in fish feeds. In striped catfish, the effects of SBL inclusion on growth performance, digestion capacity, and health status have yet to be investigated. Hence, this study aimed to determine how SBL requirements affect the growth, nutrient consumption, digestive enzyme activity, blood parameters, and antioxidant capability of striped catfish.

## 2. Materials and methods

### 2.1. Institutional ethics statement

The authors followed all applicable international, national, and/or institutional guidelines for the care and use of fish. The protocol of this study was approved by the Animal Care Use and Research Ethics Committee, Faculty of Agriculture, Kafrelsheikh University, Egypt.

### 2.2. Preparation of test diets

Five grading levels of SBL (0, 2, 4, 6, and 8%) were added to the experimental diets (Table 1). All diets were well mixed, and water was added (35–40%); then, diets were pelleted with a laboratory pelletizing machine to produce dough (2–3 mm). Afterward, pellets were kept drying at room temperature, stocked in plastic bags, and stored in the refrigerator until use [18,24]. The test diets’ chemical composition (moisture, ash, lipids, and crude protein) was confirmed using the standard method [25].

**Table 1:** Formulation and composition of the basal diet.

Ingredients	Soybean lecithin (%)				
	0	2	4	6	8
Fish meal (65%)	9	9	9	9	9
Soybean meal (45%)	41	41	41	41	41
Soybean lecithin^1^	0	2	4	6	8
Yellow corn	12	12	12	12	12
Corn gluten	6	6	6	6	6
Wheat bran	12	12	12	10	10
Wheat middling	12.92	12.92	11.92	11.92	10.92
Fish oil	4	2	1	1	0
Vitamin and mineral mix^2^	2	2	2	2	2
Dicalcium phosphate	1	1	1	1	1
Vitamin C	0.08	0.08	0.08	0.08	0.08
Total (%)	100	100	100	100	100
Chemical composition					
Crude protein (%)	30.19	30.11	30.16	30.02	30.04
Crude lipids (%)	6.76	6.86	7.09	7.59	8.29
Ash (%)	3.55	3.78	3.12	3.44	3.23
Fibers (%)	5.12	5.32	5.16	5.43	5.61
GE (MJ/kg)^3^	19.15	19.09	19.29	19.29	19.45
P/E ratio^4^	15.77	15.77	15.64	15.56	15.44

^1^Soybean lecithin (Aceitera Feneral Deheza S. A., Argentina).

^2^ The mixture of vitamins and minerals is detailed by Abdel-Latif et al. [26].

^3^Gross energy (GE) was calculated based on protein, lipid, and carbohydrate values as 23.6, 39.5, and 17.2 KJ/g, respectively [27].

^4^Protein to energy ratio (P/E) ratio (mg CP/kJ GE) = CP/GE × 1000. (kJ/100 g diet) [28–30].

### 2.3. Experimental fish

A stock of striped catfish (225 fish) was gently transported from a private farm in Kilo-21, Alexandria, Egypt, and stocked in the Laboratories and Greenhouses for the Faculties of Agriculture and Veterinary Medicine, Kafrelsheikh University, Egypt. Fish were adapted to the laboratory conditions for ten days and offered basal diets twice daily at 3% of their body weight. Subsequently, homogenized fish sizes with 8.96 ± 0.14 g initial weight were distributed for fifteen glass aquaria (80 L) representing five groups with triplicates. Each aquarium was provided with continuous aeration and stocked with 15 fish. Half of the water was exchanged every two days with fresh chlorine-free water. Fish were offered test diets twice daily up to the satiation level for 60 days. Water quality indices were regularly checked and recorded at 27.22 ± 1.1°C, 6.32 ± 0.11 mg/L, 7.76 ± 0.3, and 0.003 ± 0.001 g/L for the temperature, dissolved oxygen, pH, and total-ammonia nitrogen, respectively [28].

### 2.4. Growth indices

After 60 days, fish were starved for 24 hours, then the final weight (FW) and the number were registered for calculating the following growth performance indices:

Weight gain (WG, %) = 100 × (final weight (FW, g) − initial weight (IW, g))/IW (g); specific growth rate (SGR, %/day) = 100 × (ln FW (g)–ln IW (g))/days; feed conversion ratio (FCR) = feed intake (g)/ WG (g); protein efficiency ratio (PER) = (FW (g)–IW (g))/dry protein intake (g); survival (%) = 100 × final fish number/initial fish number.

### 2.5. Sampling

Three fish per aquarium were collected and anesthetized (Clove oil; 50 μL/L; Algomhuria Co., Egypt). Then the blood was collected from the caudal vein using 2.5 mL syringes. Serum was collected after centrifugation (3000 rpm for 5 min at 4°C) and kept at -20°C until use. Then fish were dissected, and the intestines were collected and used to analyze digestive enzyme activity. Three fish per aquarium were collected, washed, and weighed for the final body composition at -20°C. Another three fish per aquarium were dissected, and their intestines were removed and kept in formalin (10%) for the histological study.

### 2.6. Digestive enzyme activity

The homogenate was prepared by rinsing the intestines in ice-cold Phosphate-Buffered Saline (PBS) (pH 7.5; 1 g per 10 mL). It was then homogenized and centrifuged at 8000 rpm for 5 minutes, and the supernatant was collected and stored at 4°C for further analysis. The method of Lowry et al. [31] was followed to detect the total protein content. The amylase activity was estimated by following Jiang [32] and Worthington [33]. The lipase activity was analyzed following Borlongan [34] and Jin [35].

### 2.7. Biochemical analysis

According to Doumas et al. [36] and Dumas [37], total proteins and albumins were determined, while globulins content was calculated mathematically. Serum aspartate aminotransferase (AST, Ref. MX41264), alanine aminotransferase (ALT, Ref. MX41274), total cholesterol (T-CHO, Ref. MX41021), creatinine (Ref. MX1001111), uric acid (Ref. MX41001), and triglycerides (TG, Ref. MX41031) were detected by the RA-50 chemistry analyzer (Bayer) using readymade chemicals (kits) supplied by Spinreact Co. Spain, following the manufacturer’s instructions.

Superoxide dismutase (SOD, CSB-E15929Fh), glutathione peroxidase (GPx, CSB-E15930Fh), and catalase (CAT, CSB-E15928Fh) were measured using diagnostic reagent kits following the manufacturer’s (Cusabio Biotech Co., Ltd.; China) instructions. The concentration of malondialdehyde (MDA) was detected using commercial kits (Lipid peroxide (MDA), Biodiagnostic Co., Dokki, Giza, Egypt) and expressed as nmol MDA/g.

Serum lysozyme activity was determined using turbidimetric assay, according to the method described by Ellis [38] based on the lysis of *Micrococcus lysodeikticus* (Sigma, USA). Briefly, a standard suspension of 0.15 mg/mL of *M*. *lysodeikticus* was prepared in 66 mM phosphate buffer (pH 6.0). Serum (50 μL) was added to 1 mL of the bacterial suspension, and the absorbance reduction was recorded at 30-s and 4.5-min intervals at 450 nm using a spectrophotometer (SHIMADZU UV-1600PC). One unit of lysozyme was defined as a reduction in absorbance of 0.001/min.

### 2.8. Histomorphology

The histological examination was adopted according to Bancroft and Gamble [39]. The dissected intestine samples were cut into approximately 0.5 cm^3^ and fixed in neutral buffered formaldehyde 10% solution for 24 h. The samples were then dehydrated in ascending grades of alcohol, cleared with xylene, and embedded in paraffin wax. Then five μm thick sections were cut using Leica rotatory microtome (RM 20352035; Leica Microsystems, Wetzlar, Germany) and stained with hematoxylin and eosin. Two cross-sectional slices were prepared from each tissue. Finally, the tissue sections were examined using a BX50/BXFLA microscope (Olympus, Tokyo, Japan).

### 2.9. Statistical analysis

Shapiro-Wilk and Levene tests confirmed normal distribution and homogeneity of variance. The obtained data were subjected to one-way ANOVA. Differences between means were tested at *P*<0.05 level using the Duncan post-hoc test. All the statistical analyses were done via SPSS version 22 (SPSS Inc., IL, USA). The optimum soybean lecithin level was determined using polynomial regression analysis [40].

## 3. Results

### 3.1. Growth performance and carcass compositions

**Fig 1:**
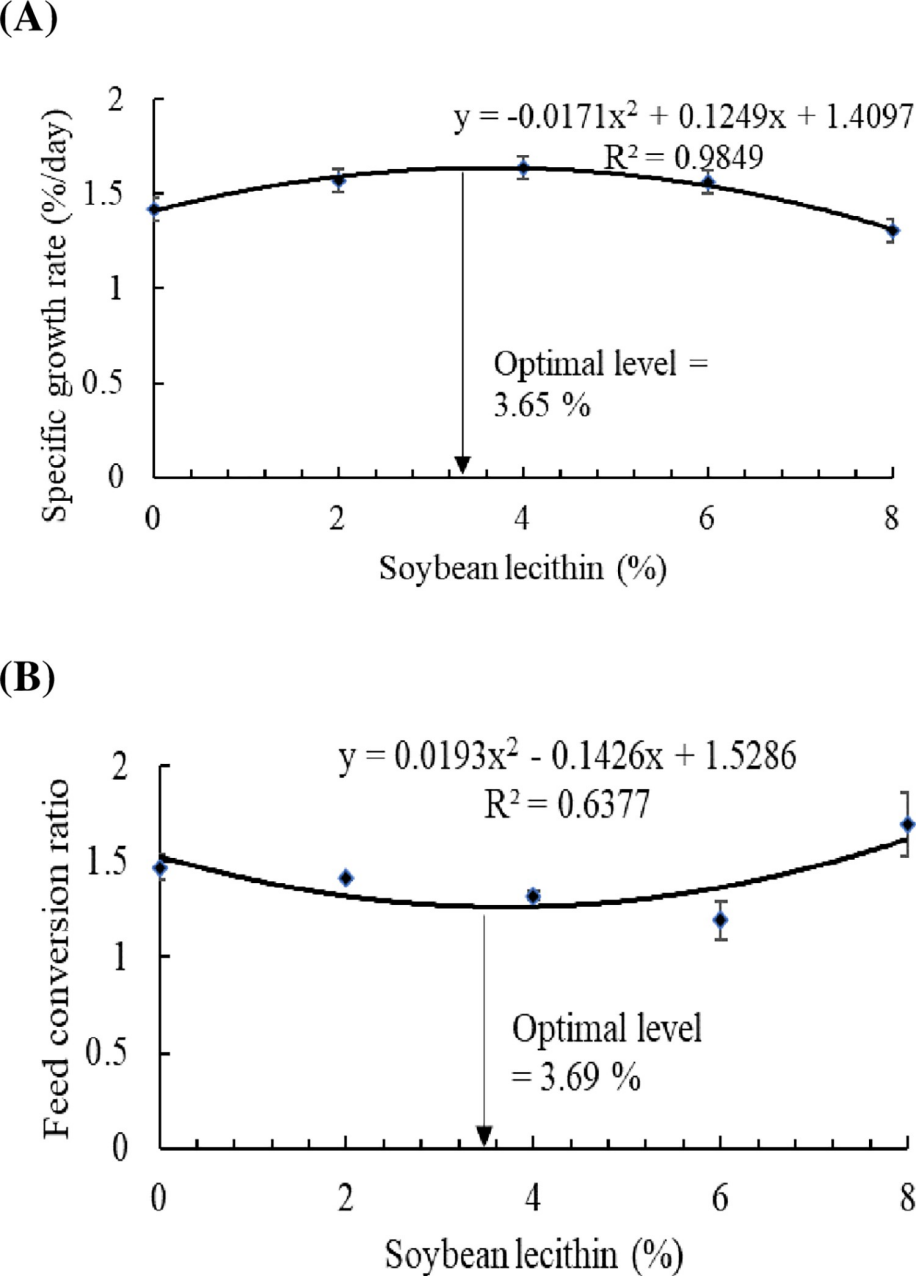
Polynomial regression analysis (*P*<0.05) between specific growth rate (A) and feed conversion ratio (B) of striped catfish fed dietary soybean lecithin for 60 days. Values are expressed as mean ±SE from triplicate groups (*n* = 9).

The FW (*P* = 0.016), WG (%) (*P* = 0.002), and SGR (*P* = 0.031) were quadratically higher in striped catfish treated with 2–4% SBL than those fed the control level (0% SBL) without significant differences (*P*˃0.05) with fish fed 6% (Table 2). The feed intake was quadratically improved in fish fed 2–4% SBL compared to 0% SBL (*P* = 0.004) (Table 2). On the other hand, the lowest FCR was in the groups of fish fed 4–6% SBL, while fish fed 2% of SBL had lower FCR than 0% SBL and quadratically higher than 4–6% SBL (*P* = 0.002). The PER was quadratically higher in fish fed 2, 4, and 6% of SBL than in fish fed 0% SBL (*P* = 0.003), with the highest PER in fish fed 6%, followed by 4% and then 2% (Table 2). The regression analysis revealed that the optimum dose of SBL is 3.65 and 3.69%, based on the results of SGR (Fig 1A) and FCR (Fig 1B).

**Table 2:** Growth performance of striped catfish fed dietary soybean lecithin for 60 days.

	Soybean lecithin (%)					*P*-value	
Item	0	2	4	6	8	Linear	Quadratic
IBW (g)	9.00±0.04	8.93±0.04	8.93±0.04	8.96±0.06	8.93±0.04	-	-
FBW (g)	21.11±0.29^b^	22.89±0.35^ab^	23.89±0.22^a^	22.89±0.97^ab^	19.56±0.66^b^	-	0.016
WG (%)	134.57±3.09^b^	156.22±3.89^ab^	167.42±2.73^a^	155.57±10.63^ab^	118.92±7.42^b^	-	0.002
SGR (%/day)	1.42±0.02^b^	1.57±0.03^ab^	1.64±0.02^a^	1.56±0.07^ab^	1.30±0.06^b^	-	0.031
FI (g/fish/60 days)	17.80±0.57^b^	19.74±0.46^a^	19.79±0.42^a^	16.48±0.59^b^	17.79±0.77^b^	-	0.004
FCR	1.47±0.07^a^	1.41±0.00^b^	1.32±0.02^c^	1.20±0.10^c^	1.70±0.17^a^	-	0.002
PER	1.94±0.09^d^	2.01±0.00^c^	2.16±0.04^b^	2.64±0.11^a^	1.88±0.11^d^	-	0.003
Survival (%)	100.00±0.00	100.00±0.00	100.00±0.00	100.00±0.00	100.00±0.00	-	-

*Means ± SE (*n* = 3) with different letters show significant differences (*P*<0.05). IBW: Initial body weight, FBW: Final body weight, WG: Weight gain, SGR: Specific growth rate, FI: Feed intake, FCR: Feed conversion ratio, PER: Protein efficiency ratio.

The carcass protein content was quadratically higher (*P* = 0.043) in striped catfish treated with SBL compared to 0% SBL (Table 3). The carcass lipid content was also quadratically higher (*P* = 0.038) in fish fed 2–4% SBL than in fish fed 0, 6, and 8% (Table 3). No effects for SBL were observed on the moisture and ash contents in the carcass of striped catfish (*P*˃0.05).

**Table 3:** Carcass composition of striped catfish fed dietary soybean lecithin for 60 days.

Item	Soybean lecithin (%)					*P*-value	
	0	2	4	6	8	Linear	Quadratic
Moisture (%)	75.95±0.35	74.70±0.36	74.46±0.10	74.81±0.24	75.01±0.48	-	-
Crude protein (%)	13.40±0.12^b^	14.74±0.25^a^	14.53±0.27^a^	14.34±0.15^a^	14.49±0.11^a^	-	0.043
Ether extract (%)	5.57±0.05^b^	6.27±0.14^a^	6.22±0.18^a^	5.84±0.14^b^	5.70±0.19^b^	-	0.038
Ash (%)	4.49±0.19	4.20±0.09	4.26±0.22	4.22±0.10	4.42±0.06	-	-

*Means ± SE (*n* = 3) with different letters show significant differences (*P*<0.05).

### 3.2. Digestive enzyme activity

The lipase activity was quadratically higher in fish fed 2, 4, and 6% of SBL than in fish fed 0% SBL (*P* = 0.027), with the highest activity in fish fed 4%, followed by 2 and 6% (Table 4). The amylase activity was quadratically higher in fish fed 2–4% SBL than in fish fed 0% SBL (*P* = 0.033) (Table 4). The protease activity was quadratically higher in fish fed 2, 4, and 6% of SBL than in fish fed 0% SBL, while fish fed 2 and 4% had higher protease activity than those fed 6% (*P* = 0.042) (Table 4).

**Table 4:** Digestive enzyme activity of striped catfish fed dietary soybean lecithin for 60 days.

Item	Soybean lecithin (%)					*P*-value	
	0	2	4	6	8	Linear	Quadratic
Lipase activity (unit/mg protein)	18.06±0.13^c^	20.53±0.52^b^	22.49±0.58^a^	20.47±0.67^b^	18.06±0.42^c^	-	0.027
Amylase activity (unit/mg protein)	20.02±0.38^b^	24.30±0.25^a^	24.26±0.21^a^	21.38±0.62^b^	20.09±0.14^b^	-	0.033
Protease activity (unit/mg protein)	17.45±0.42^c^	20.35±0.39^a^	21.82±0.15^a^	19.49±0.56^b^	17.43±0.35^c^	-	0.042

*Means ± SE (*n* = 3) with different letters show significant differences (*P*<0.05).

### 3.3. Intestinal histology

**Fig 2:**
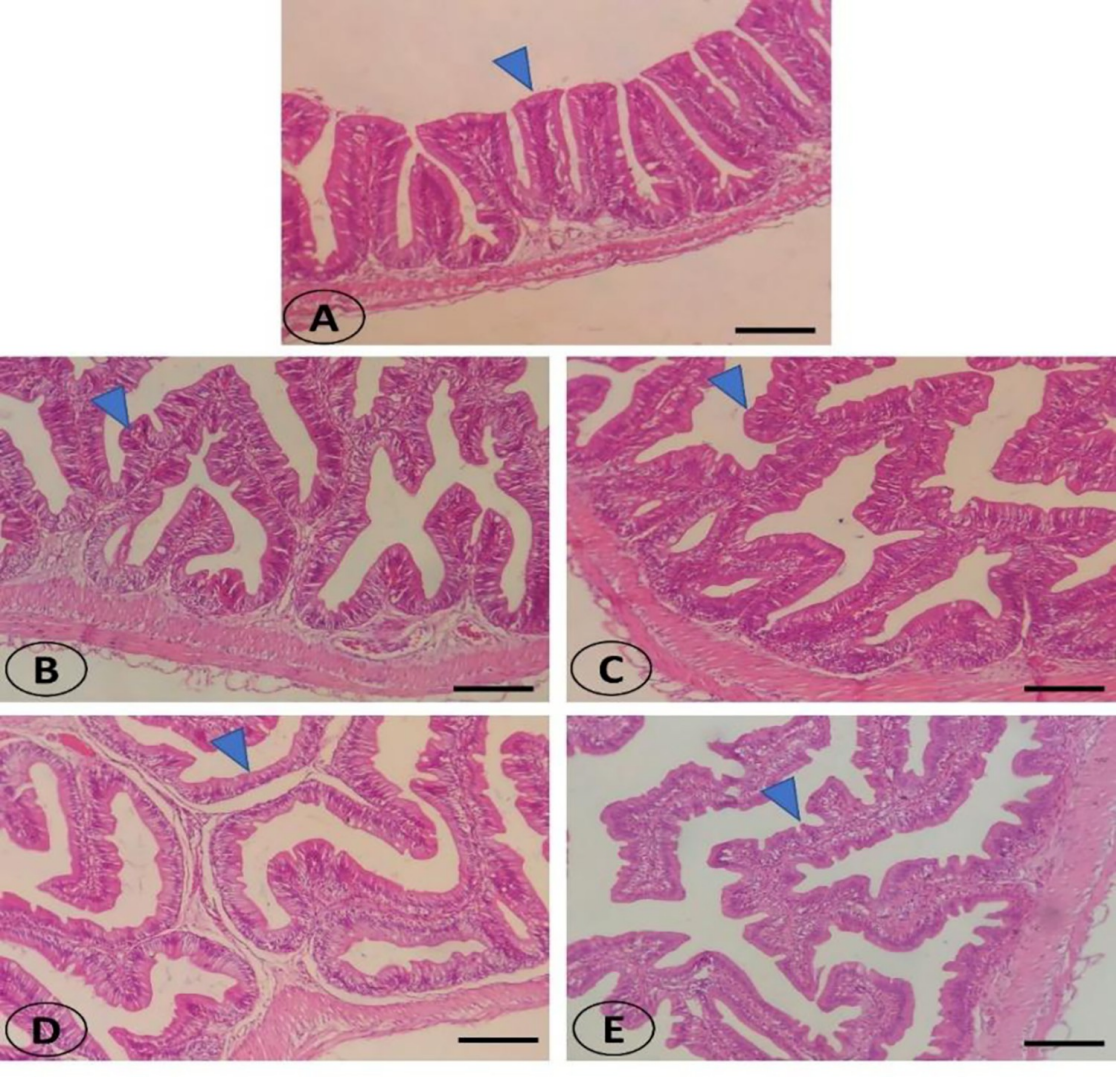
Histomorphology of the middle intestine of striped catfish in the control fish (A; 0%) and gradually increased levels of soybean lecithin (B, C, D, E; 2, 4, 6, 8% respectively) showing evident enrichment and branching of intestinal villi (blue arrowhead) by increased levels of soybean lecithin in the supplemented diet. Stain H&E. Bar = 100 μm.

The intestine of SBL non-supplemented fish revealed a normal intact intestinal wall and intestinal villi (Fig 2A). The intestinal wall is covered externally by tunica serosa and lined internally by mucosa and the muscular layer. The intestinal villi were mainly formed of simple columnar enterocytes with an absorptive brush border around the connective tissue core. Few goblet cells were found between the enterocytes. The gradually increased levels of SBL improved the structural appearance and increased the intestinal villi length and branching appearance (Fig 2B–2E).

### 3.4. Biochemical parameters

TG and T-CHO levels were only the blood biomarkers affected by SBL inclusion in striped catfish diets, while the remaining biomarkers were not significantly different (*P*˃0.05) (Table 5). The TG (*P* = 0.021) and T-CHO (*P* = 0.002) were linearly increased in fish fed 4, 6, and 8% than in fish fed 0% SBL, with the highest being in fish fed 8%.

**Table 5:** Blood biochemical indices of striped catfish fed dietary soybean lecithin for 60 days.

Item	Soybean lecithin (%)					*P*-value	
	0	2	4	6	8	Linear	Quadratic
ALT (U/I)	3.17±0.09	3.15±0.06	3.14±0.04	3.12±0.06	3.21±0.06	-	-
AST (U/I)	71.23±0.64	70.46±0.41	70.94±0.36	71.40±0.53	71.65±0.57	-	-
Total protein (g/dl)	4.32±0.05	4.57±0.12	4.62±0.07	4.69±0.07	4.27±0.08	-	-
Albumin (g/dl)	2.35±0.06	2.56±0.08	2.33±0.18	2.59±0.06	2.52±0.17	-	-
Globulin (g/dl)	1.97±0.08	2.01±0.07	2.29±0.17	2.09±0.13	1.75±0.25	-	-
Creatinine (mg/dl)	0.25±0.01	0.23±0.01	0.25±0.01	0.24±0.01	0.24±0.01	-	-
Uric acid (mg/dl)	1.83±0.03	1.84±0.09	1.83±0.06	1.81±0.05	1.80±0.04	-	-
T-CHO (mg/dl)	79.48±0.40^c^	82.14±0.68^c^	89.45±0.47^b^	90.40±0.44^b^	102.25±0.22^a^	0.002	-
TG (mg/dl)	125.48±1.55^c^	134.84±1.59^c^	146.23±1.98^b^	155.88±1.73^b^	206.13±0.65^a^	0.021	-

*Means ± SE (*n* = 3) with different letters show significant differences (*P*<0.05). ALT: Alanine aminotransferase, AST: Aspartate aminotransferase, T-CHO: Total cholesterol, TG: Triglycerides.

### 3.5. Immune indices

No effects for SBL were observed on the total protein, albumin, and globulin of striped catfish (*P*˃0.05) (Table 5). The lysozyme activity was quadratically higher in fish fed 2, 4, and 6% of SBL than in fish fed 0% SBL, with higher activity in fish fed 2 and 4% than 6% (*P* = 0.002) (Table 6).

**Table 6:** Blood immunity and antioxidative responses of striped catfish fed dietary soybean lecithin for 60 days.

Item	Soybean lecithin (%)					*P*-value	
	0	2	4	6	8	Linear	Quadratic
Lysozyme activity (unit/ml)	34.30±0.69^c^	36.30±0.62^a^	36.97±0.15^a^	35.42±0.36^b^	33.67±0.32^c^	-	0.002
Superoxide dismutase (IU/L)	24.00±0.72^c^	27.22±0.55^b^	29.45±0.35^a^	29.25±0.58^a^	25.46±0.31^c^	-	0.034
Catalase (IU/L)	34.28±0.53^c^	36.58±0.40^b^	39.02±0.56^a^	39.35±0.60^a^	34.49±0.40^c^	-	0.011
Glutathione peroxidase (IU/L)	24.12±0.54^c^	27.74±0.27^b^	29.28±0.46^a^	30.33±0.41^a^	26.34±0.75^b^	-	0.042
Malondialdehyde (nmol/g)	22.30±0.63^a^	21.00±0.35^a^	18.40±0.47^b^	18.31±0.58^b^	21.37±0.24^a^	-	0.034

*Means ± SE (*n* = 3) with different letters show significant differences (*P*<0.05).

### 3.6. Antioxidant and oxidant indices

SOD (*P* = 0.034) and CAT (*P* = 0.011) were quadratically higher in fish fed 2, 4, and 6% of SBL than in fish fed 0% SBL, with higher activity in fish fed 4 and 6% than 2% (Table 6). The GPx was quadratically higher in fish fed 2, 4, and 6% of SBL than in fish fed 0% SBL, with higher activity in fish fed 4–6% SBL SBL than in 2 and 8% (*P* = 0.042) (Table 6). The MDA level was quadratically lower in fish fed 4–6% SBL than in fish fed 0% SBL (*P* = 0.034) (Table 6).

## 4. Discussion

Dietary soybean lecithin (SBL) enhanced the productivity and well-being of finfish species [12,13]. SBL can enhance digestion capacity, palatability, and feed consumption in carnivorous, omnivorous, and herbivorous fish habitats [41]. Concisely, this study indicated that striped catfish treated with SBL had enhanced growth performance. Also, improved growth performance was shown in Caspian brown trout [19], common carp [42], gilthead seabream [20], Nile tilapia [21], and Dojo loach [43]. However, channel catfish (*Ictalurus punctatus*) fed dietary SBL showed no changes in growth performance [44]. The differences can be attributed to fish sizes and experimental conditions. SBL is a rich source of phospholipids needed to synthesize lipoproteins that facilitate the lipid’s transportation through the intestinal layers to the entire body tissues and fluids [11]. Hence the lipid deposition and available energy required for the growth increase [45]. The increased growth rate can be related to improved feed intake and utilization of SBL addition [46]. Indeed, the study showed a reduced FCR in striped catfish with SBL feeding. Similarly, channel catfish fed SBL at 4% had improved FCR [44]. The improved FCR reflects enhanced feed utilization in striped catfish, which can be related to enhanced feed intake and digestion. It has been stated that SBL effectively increases lipoproteins formation involved in the digestion of lipids and the absorption of nutrients in fish intestines [15].

Feed utilization and feed intake are strongly related to the activation of digestive enzymes in the intestines of fish [5]. Lipase, amylase, and protease are involved in the digestion of lipids, carbohydrates, and proteins in fish diets [47]. This study showed enhanced amylase, lipase, and protease enzymes in striped catfish treated with soybean lecithin, especially with a 4% inclusion level. In line with the obtained results, Caspian brown trout [19], silvery-black porgy (*Sparidentex hasta*) [48], and common carp [42]-fed SBL showed enhanced digestive enzyme activity. SBL can be hydrolyzed in fish intestines and help in lysophosphatidylcholine formation associated with the energy conservation required for the biosynthesis and activation of digestive enzymes [20,49].

The gradually increased levels of SBL improved the structural appearance and increased the intestinal villi length and branching appearance in striped catfish. The results are similar to Jenabi Haghparast et al. [19], and El-Sayed et al. [21], who indicated that Caspian brown trout and Nile tilapia fed dietary SBL had enhanced intestinal histological features. Epithelial thickness and villus length increased in striped catfish, indicating the intestinal surface area and absorption capability by dietary SBL [50]. The abundance of phospholipids in SBL may enhance the intestinal histological features of striped catfish. Phospholipids are involved in synthesizing lipoproteins required for the structure of body tissues and achieving several physiological functions [11]. It is more apparent that SBL enhanced the growth performance of striped catfish. Based on the obtained results, the improved FCR, digestive enzyme activity, and intestinal histological features are the main reasons for the enhanced growth performance of striped catfish [51,52]. The role of phospholipids on the activation of flux rate through the cell membrane enterocytes, thereby facilitating the permeability of digested macromolecules [18,53]. Consequently, efficient feed utilization and absorption resulting from phospholipids lead to high growth performance.

The results also showed that fish fed SBL had enhanced lipid and protein contents in the carcass. The results are similar to Sink and Lochmann [44], who indicated that channel catfish fed SBL had enhanced carcass protein and lipid contents. Further, El-Sayed et al. [21] stated that Nile tilapia treated with SBL showed increased carcass protein and lipid contents. High protein content can be related to the role of lecithin, rich in phospholipids, in reducing the energy needed for the biosynthesis of phospholipids [54]. In addition, increased lipid content results from the depletion of digested and absorbed lipids from the intestines due to SBL feeding [55]. The increased PER in striped catfish fed dietary SBL may explain the increased crude protein level. Indeed, high PER refers to the efficient protein accumulation in the carcass and, thereby, a high protein level.

It is necessary to correlate the nutritional value of feed and fish’s physiological and immunological status [56,57]. Well-formulated feeds that consider the sufficiency of essential nutrients, including lipids, for the requirements of fish are the main guarantee for fish’s stable metabolic and physiological performances [58,59]. In this regard, we evaluated the biochemical blood index of striped catfish fed soybean lecithin. Under the current trial conditions, no significant effects were seen on the blood proteins, creatinine, urea, ALT, and AST. However, the TG and T-CHO levels were increased by dietary soybean lecithin. The results are in line with Jenabi et al. [19], who reported increased blood cholesterol levels in Caspian brown trout-fed dietary soybean lecithin. Further, channel catfish fed dietary SBL showed increased blood cholesterol [44]. The increased blood cholesterol and triglycerides are probably related to the role of SBL in regulating the synthesis and transportation of lipids [19], including cholesterol and triglycerides. In this regard, Taghavizadeh et al. [18] reported that rainbow trout-fed lysophospholipids had increased cholesterol and HDL while decreasing LDL. Phospholipids may act as emulsifiers to reduce bile salts and low cholesterol secretion [53], thereby high accumulation levels in the entire body. Enhancing lipid absorption from intestinal cells resulted in a greater lipid transport rate and metabolism rate in the liver, which raised cholesterol levels [10]. On the other hand, the absence of changes in the blood proteins, creatinine, urea, ALT, and AST confirm the stable metabolic rates and physiological condition of striped catfish fed dietary SBL [60].

Fish farming faces several biotic and abiotic stressors interrupting fish’s metabolic, physiological, and immunological responses [61]. Oxidative stress also hits fish and may weaken fish immunity and lead to the high availability of infection [62]. The high release of reactive oxygen species (ROS) oxidizes lipids and induces oxidative stress, causing tissue damage [63]. Also, high oxidative stress refers to high lipid peroxidation, expressed as MDA level [64]. Fish develop several enzymatic antioxidative responses to relieve the impacts of MDA, such as SOD, CAT, and GPX [65]. In this study, striped catfish fed SBL (2–6%) showed increased SOD, CAT, and GPX while reducing the MDA level. The results agree with Jenabi et al. [19], Adel et al. [42], Saleh et al. [20], and Gao et al. [43], who reported that Caspian brown trout, common carp, gilthead seabream, and Dojo loach had activated antioxidative status due to soybean lecithin. The enhancement of the antioxidative status is related to reducing lipid peroxidation under the effect of lecithin phospholipids [66]. Besides, the enhanced antioxidative response is also associated with increased cell viability and metabolic status [18]. Further, Taghavizadeh et al. [18] concluded that rainbow trout-fed lysophospholipids showed enhanced antioxidative response due to the activation of antioxidative enzymes involved in releasing free radicals. Another possible reason is related to the effect of phospholipids in the SBL to upregulate the endogenous antioxidant gene expression in fish [18].

Balanced feed formulations can also enhance the immunity of fish and, thereby, resistance to pathogenic invaders [56]. In this regard, we analyzed the lysozyme activity in the blood of striped catfish fed soybean lecithin. The results indicated improved lysozyme activity in fish fed 2–6% soybean lecithin. The results are similar to El-Sayed et al. [21], who indicated that Nile tilapia-fed SBL had enhanced lysozyme activity. The enhancement of lysozyme activity is probably related to the role of phospholipids in improving cell permeability and fluidity and, thereby, immunity. Further, dietary SBL balances the fatty acid content in the fish body and regulates metabolic and physiological functions.

The results indicated that SBL could be included in striped catfish at 3.65–4.42% of the feed formulation. The inclusion levels were calculated using polynomial regression analysis based on the SGR, FCR, lipase activity, and SOD under the current trial conditions. The calculated level of supplementation is similar mainly to Jenabi et al. [19], who suggested that SBL can be included at 6–9% in the diets of Caspian brown trout. Further, SBL is recommended to channel catfish at 4–4.3%. Adel et al. [42] reported that common carp require 2–3% SBL for optimum growth performance and physiological status. In golden mahseer, SBL is recommended up to 2% for enhancing growth performance and health status [17]. The determination of optimum levels of SBL is also reported in other studies; however, these levels differ based on the fish species, life stage, rearing conditions, and diet composition.

## 5. Conclusions

In summary, SBL can enhance the feed consumption and growth performance of striped catfish. Further, SBL improved the structural appearance and increased the intestinal villi length and branching appearance as well as the digestive enzyme activities resulting in enhanced digestion capacity. Subsequently, fish showed improved immune and antioxidative responses. The overall results indicated that SBL could be included at 3.65–4.42% for successful fingerling farming.

## Supporting information

S1 Dataset (XLSX)
